# Lip Repositioning Surgery: A Simple Smile and Life Transformation Procedure

**DOI:** 10.1155/2024/6156806

**Published:** 2024-02-26

**Authors:** Besir Salihu, Zana Agani, Arta Sinanaj Demiri

**Affiliations:** ^1^University of Prishtina, Faculty of Medicine, Public Health Department, Pristina, Kosovo; ^2^Private Polyclinic “Aesthetica”, Mitrovicë, Pristina, Kosovo; ^3^Department of Oral Surgery, Medical Faculty of Prishtina, University of Prishtina, Pristina, Kosovo

## Abstract

Excessive display of the gums when smiling, known as gummy smiles, can lead to aesthetic concerns and emotional discomfort for individuals. In recent times, there has been a notable rise in the popularity of gummy smile surgery as a cosmetic procedure aimed at addressing this problem. A novel technique known as lip repositioning has emerged and gained recent popularity, either on its own or in conjunction with other approaches. In specific situations, it offers a simpler, less intricate alternative to more extensive surgical procedures, resulting in a pleasing and satisfactory camouflage effect while reducing postoperative complications. This case report illustrates the effective treatment of a gummy smile in a patient with excessive gum exposure using a lip repositioning technique. The procedure involved the removal of a partial thickness strip of mucosa lining in the maxillary buccal vestibule, without including the labial frenulum, and suturing the lip lining to the gum line. Our aim was to present lip repositioning as an effective method for reducing excessive gum exposure and addressing any unevenness in a minimally invasive manner. We sought a solution that offers long-lasting results over time while minimizing the chances of the issue recurring, with a 14-month follow-up.

## 1. Introduction

A gummy smile is a common cosmetic concern that can negatively impact someone's social interactions and self-confidence [1]. It is characterized as the exposure of a substantial piece of the upper gum while smiling [2]. In order to address this problem, gummy smile surgery has become a feasible option. It uses a variety of procedures to adjust gingival presentation and create a harmonious smile [3, 4].

Understanding the origin of gummy smile is crucial to selecting the appropriate course of treatment [5]. Many reasons, such as improper tooth eruption, excessive maxillary vertical development, hyperactive upper lip muscles, and insufficient lip length, can result in gummy smiles [5, 6].

With a special focus on the lip repositioning approach, this case report provides a successful management of gummy smile. In general, crown lengthening, orthognathic surgery, and botulinum toxin injections can be classified as additional procedures [7], advantages and disadvantages of which are described in Table 1.

It is crucial to remember that not everyone is a good candidate for gummy smile surgery. The underlying cause for the gummy smile and the person's general dental health are among the variables that determine the procedure's eligibility.

Lip repositioning is contraindicated in cases with substantial vertical maxillary excess and minimal zone of attached gingiva, which can complicate flap design, stabilization, and suturing [8, 9].

In this case report, we aimed to showcase lip repositioning as an efficient approach to decrease excessive gum exposure and rectify asymmetry with minimal invasiveness. Our goal was to find a solution ensuring enduring outcomes while reducing the likelihood of the problem reoccurring, as evidenced by a 14-month follow-up period.

## 2. Case Presentation

**Figure 1:**
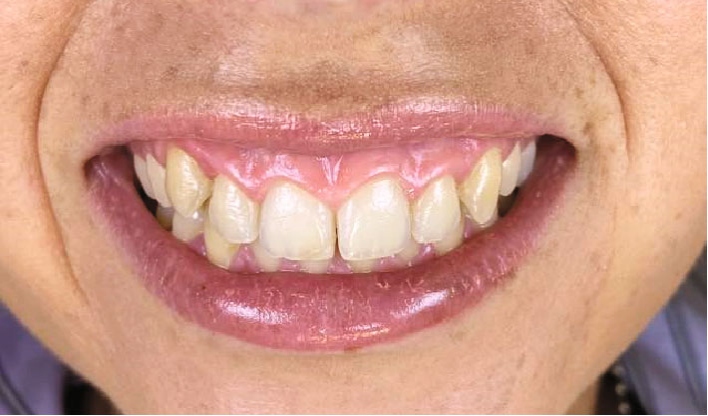
Preoperative photography.

The 27-year-old female patient, two months before her wedding, presented with aesthetic complaints of gingival display while smiling (Figure 1), a complaint that has followed her throughout her life.

The patient's medical history did not indicate any systemic disease or other health issues.

Upon clinical examination, excessive gingival display over 4 mm was shown, with normal maxillary anterior anatomic proportions. Short clinical crowns with moderate gingival phenotype are also observed.

During probing, gingival pockets are detected, while examination using the visual method reveals adequate gingival attachment.

Her orthodontist reports positive history of class II malocclusion, treated with mobile appliances during childhood, which could be one of the etiological factors of this gummy smile.

**Figure 2:**
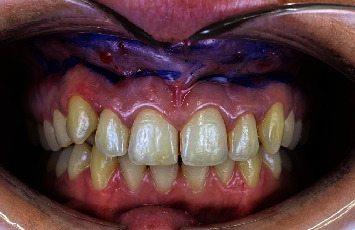
Incision lines marked.

The smile line, which measured about 16 mm, was checked using preoperative measurements (Figure 1). From maxillary right to left second premolar, a local anesthetic (Xylocaine 2% with adrenaline, 1 : 100,000, and epinephrine, 1 : 50,000) was injected into the vestibular mucosa and lip. The incisions on the dried tissues were marked with a marking pencil (Figure 2).

Raising of a partial thickness flap was performed, with the first incision at the level of mucogingival junction; starting from second premolars on both sides, the second incision was made on the labial mucosa, approximately 10 to 12 mm apart from the first one with labial frenulum preservation.

**Figure 3:**
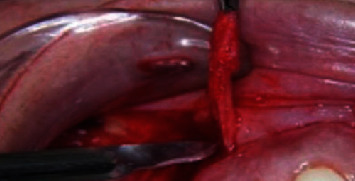
Epithelium layer removal.

**Figure 4:**
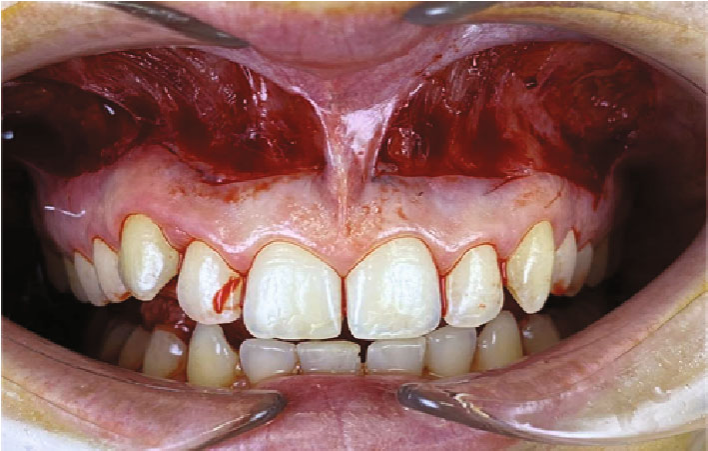
Exposed connective tissue.

The incisions were connected at each second premolar and very close to the labial frenulum, creating two elliptical outlines of the incisions. The epithelium layer was removed (Figure 3), exposing the connective tissue underneath (Figure 4).

Resorbable sutures were used to stitch the flap edges after the strip of connective tissue was removed.

**Figure 5:**
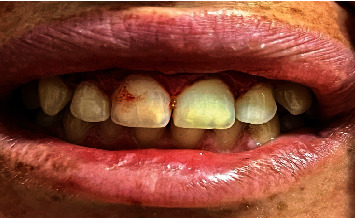
Postoperative picture after gingivectomy, presenting with upper lip swelling.

Gingivectomy was also performed (Figure 5) regarding to the conventional method without osteotomy, using the Sanders gingivectomy scalper. The goal was to enhance the shape and contour of the gum tissue following gummy smile surgery. In this step, any residual unevenness or inconsistencies in the gumline were rectified.

Antibiotics (amoxicillin 500 mg tid for 5 days) and analgesics (ibuprofen 650 mg tid for 4 days) were prescribed as part of the postoperative guidelines. Cold compresses to reduce swelling were also preferred.

In addition, the patient was instructed to minimize their speaking and smiling in order to avoid making active lip movements and refraining from brushing the surgical region for a full week.

The surgical site was irrigated with saline throughout the first week of follow-up, and there were no biological problems. However, during the first 10 days, the patient reported burning pain, lip swelling, and discomfort while speaking. These complaints went away completely with the passage of time.

**Figure 6:**
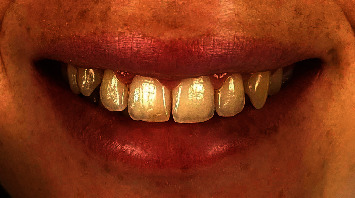
Postoperative check-up three weeks after the operation.

**Figure 7:**
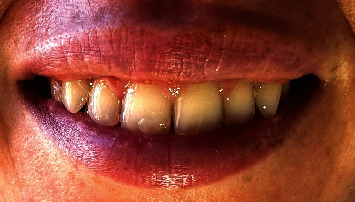
After a 14-month postsurgery follow-up, there is no recurrence observed, and signs of interdental papilla regrowth have emerged between the first and second right upper incisors.

The postoperative results were aesthetically satisfactory, three weeks after the intervention (Figure 6) and 14-month follow-up after the surgery (Figure 7). The emotional impact according to the patient was impeccable.

## 3. Discussion

The technique of surgical lip repositioning remains a successful technique either alone or in combination with other techniques [10]. The goal remains the correction of the gummy smile, and based on many studies [11–13] and our clinical experience in the case presented above, this technique gives promising results.

In this case, we preserved the labial frenulum since there was no indication for frenectomy. Based on a meta-analysis by Younespour et al., the most effective gummy smile reduction with the lip repositioning technique remains in cases where the frenulum is not involved [14]. However in cases where the labial frenulum is situated in close proximity to the gum line, it may cause an excessive upward pull on the upper lip during smiling, causing a gummy smile. In these situations, lip repositioning surgery combined with labial frenectomy can aid by moving the frenulum into a more desirable position [15, 16].

There are countless cases [17–19] when the combination of gingivectomy with lip repositioning is done, especially in cases where we have more than 4 mm of gingival exposure [9], as in our case. Therefore, performing a gingivectomy following lip repositioning surgery helps eliminate any remaining irregularities or inconsistencies.

Lip repositioning can potentially enhance gum aesthetics by 3-4 mm according to Tawfik et al. [20].

The biggest struggle in the selection of the surgical technique for gummy smile correction remains in the technique which gives the least relapses. AlJasser evaluated the modified technique with the placement of periosteal sutures as more effective against relapses [21].

Narayan and Rajasekar [22] in their study involving 373 patients explored various gingivectomy techniques. They reached the conclusion that approaches incorporating osteotomy, specifically surgical crown lengthening with a modified Widman flap and an apically repositioned flap, result in less relapse when compared to traditional techniques, such as the one employed in our case.

Although the aforementioned methods have demonstrated greater efficacy in preventing recurrences, we have opted to proceed with a traditional gingivectomy without osteotomy. Following the periodontal assessment, we determined the absence of infrabony pockets or insufficient gingival attachment.

According to various studies [23–25], a flap procedure and bone recontouring are necessary only in cases where there is inadequate attached gingiva and infrabony pockets.

The primary indication for this surgical procedure was purely aesthetic, due to periodontal parameters aforementioned.

Additionally, the patient selected the simplest procedure after receiving explanations about all available methods.

To avoid the lip muscle reverting to its initial position after lip repositioning surgery, some publications [26, 27] suggest detaching the muscle insertion. This action can potentially alleviate tension on the flap during the suturing process.

However, recidivism is sometimes inevitable and can happen for several reasons such as natural aging, issues with the healing process, technique and skill, and individual variability as well [28].

As per a systematic review and meta-analysis conducted by Dos Santos-Pereira et al. [7], an estimated 25% relapse could occur within a year. To enhance predictability and stability, practitioners are advised to integrate the procedure with additional methods like plastic periodontal surgeries, restorative procedures, or the use of botulinum toxin injections.

A 14-month follow-up in our case showed no relapses, with signs of interdental papilla regrowth between the first and second right upper incisors; however, a further more follow-up is needed.

## 4. Conclusion

Lip repositioning surgery remains a gold standard for patients who want to correct the gummy smile. The long-lasting outcomes, without very high costs and with the simplicity of the procedure, make this technique superior to other techniques.

However, the combination of many techniques, whether in the presented case or in the browsed literature, improves every defect of lip repositioning surgery.

A careful case selection is the key, to provide good results and also to prevent undesired outcomes. Whether to compare or to combine, the selection of the right technique should be based on the technique that gives fewer complications and relapses.

For a comprehensive long-term assessment, additional studies will be required to gauge the stability and efficacy of this technique.

## Figures and Tables

**Table 1:** Treatment options for gummy smile.

Treatment modality	Description	Advantages	Disadvantages
Botox injections	Injection of botulinum toxin to the muscles of the upper lip in order to minimize gum exposure.	Minimum downtime, noninvasive, and transient results.	In severe cases, there may be limited effectiveness, numerous sessions required, and possibly short-lived results.
Crown lengthening	Surgery to reveal more of the tooth crown by removing extra gum tissue.	Long-lasting outcomes, suitable for moderate to severe situations.	Invasive process, risk for postoperative discomfort, extended recovery time.
Lip repositioning	Surgical modification of the upper lip to restrict movement and hide the gums.	Long-lasting outcomes, particularly in severe situations.	Invasive, may cause asymmetry in the lips, take longer to heal, and need a skilled surgeon.
Orthodontic treatment	Aligning the upper jaw and realigning teeth using braces or clear aligners.	Long-term benefits for oral health; long-term drawbacks.	Needs time commitment, potential case limitations, and lack of stand-alone treatment.
Gingival contouring	Reshaping gum tissue with a scalpel or laser to achieve a more harmonious ratio of gum to tooth.	Quick process with minimal discomfort.	There is a chance of gum sensitivity, a range of results, and suitability for certain situations.
Surgical jaw correction	Orthognathic surgery is aimed at lessening excessive gum exposure by realigning the upper jaw.	Addresses underlying skeletal problems and provides a complete treatment.	Extremely invasive, has a long recovery period, and calls for skilled surgeons.
